# Screening for Chronic Kidney Disease by Mobile Health Unit Outreach

**DOI:** 10.1001/jamanetworkopen.2026.2312

**Published:** 2026-03-18

**Authors:** Robert D. Brook, Steven J. Korzeniewski, Bethany Foster, Paul Kurian, Brian Reed, Anna Steinberg-Abreu, James L. Young, Phillip D. Levy

**Affiliations:** 1 Integrative Biosciences Center, Wayne State University, Detroit, Michigan; 2 Division of Cardiovascular Disease, Department of Internal Medicine, Wayne State University, Detroit, Michigan; 3 Department of Emergency Medicine, Wayne State University, Detroit, Michigan

## Abstract

This cross-sectional study reports population-wide screening results and risk factors for chronic kidney disease among individuals who received screening from mobile health units in the Detroit, Michigan, area.

## Introduction

Guidelines advocate chronic kidney disease (CKD) screening approaches that target individuals with risk factors (eg, diabetes).^1^ However, recent studies suggest that population-wide screening for CKD may improve public health and could be cost-effective with the advent of effective preventive treatments (eg, sodium-glucose transport-2 inhibitors).^2,3^ Greater benefits, particularly at younger ages, were shown for non-Hispanic Black populations with increased CKD-related burden.^2^ Nonetheless, proactive large-scale screening systems are uncommon, even in high-risk socially disadvantaged communities such as Detroit, Michigan.^3^ To improve earlier detection of cardiovascular-kidney-metabolic risk factors, we developed a population health program that deploys mobile health units (MHUs) across metropolitan Detroit.^4^ The goal is to decrease area-wide morbidity and mortality and reduce health disparities. We report program findings regarding population-wide CKD screening results and risk factors.

## Methods

This cross-sectional study, conducted from July 7, 2022, to August 24, 2025, was approved by Wayne State University’s institutional review board and is reported in accordance with the STROBE reporting guideline. The institutional review board determined that the study met criteria for waiver of informed consent because the research involved minimal risk to participants and could not practicably be conducted without the waiver. We analyzed a deidentified limited use dataset provided under an agreement between Wayne State University and Wayne Health. The MHU program has been previously described.^4^ Briefly, 5 to 7 MHUs deploy with nonphysician staff 5 to 6 days per week to partner locations across metropolitan Detroit. After a brief medical history was taken, adults 18 years or older were offered screenings for high blood pressure (BP), diabetes (ie, hemoglobin A_1c_ [HbA_1c_] measurement), and CKD (ie, estimated glomerular filtration rate [eGFR] calculated from a comprehensive metabolic panel). Seated blood pressure was measured onsite after 5 minutes of rest using the mean of 3 upper arm readings (1-minute intervals) determined by an Omron 907XL monitor (Omron Healthcare).^5^ Venous blood samples were obtained onsite and shipped to a partner laboratory (Quest Diagnostics or Labcorp). We analyzed results from July 7, 2022, to August 24, 2025, after the Chronic Kidney Disease Epidemiology Collaboration (CKD-EPI) 2021 creatinine equation for calculating eGFR using creatinine (eliminating race-based adjustments to enhance equity) was adopted.

We estimated relative risk with 95% CIs for levels of CKD using log-binomial generalized estimating equations models that accounted for repeated measurements by clustering on patient identification numbers using an exchangeable working correlation matrix and robust (sandwich) standard errors. Multivariable adjusted models included patient characteristics selected a priori. Type I error was limited to 5% (2-sided α = .05), and analyses were performed using SAS, version 9.4.

## Results

Results from our cohort of 5128 unique patients with 5973 encounters are shown in Table 1; 2293 patients (44.7%) had eGFRs of 60 to 89 mL/min/1.73 m^2^, whereas 579 (11.3%) had CDK stage 3 or higher (eGFR <60 mL/min/1.73 m^2^). Older age, Black race, and diabetes (HbA_1c_ ≥6.5%) were associated with higher risks for CKD stage 3 or higher (Table 2).

**Table 1.** Characteristics of Patients at Mobile Health Unit Encounters^a^

Kidney disease screening cohort	Unique patients (N = 5128)^b^	Total encounters (N = 5973)
Age, median (IQR), y [range, 18-103 y]	56 (41-67)	57 (42-68)
Race and ethnicity, No. (%)^c^		
American Indian or Alaska Native	11 (0.2)	17 (0.3)
Asian	126 (2.5)	159 (2.7)
Black or African American	3560 (69.4)	4175 (69.9)
Middle Eastern or North African	10 (0.2)	10 (0.2)
White	732 (14.3)	845 (14.2)
Other not specified	393 (7.7)	427 (7.1)
Patient declined	293 (5.7)	337 (5.6)
Sex, No. (%)		
Female	3150 (61.4)	3684 (61.7)
Male	1978 (38.6)	2289 (38.3)
Systolic BP, median (IQR), mm Hg (range, 80-231 mm Hg)	121 (111-133)	121 (111-133)
Diastolic BP, median (IQR), mm Hg (range, 34-146 mm Hg)	75 (67-83)	75 (67-83)
Systolic BP ≥130 mm Hg, No. (%)	1380 (30.9)	1634 (31.5)
Laboratory test results		
HbA_1c_, median (IQR), % (range, 4.2%-15.4%)	5.8 (5.4-6.2)	5.8 (5.5-6.2)
HbA_1c_ ≥6.5%, No. (%)	811 (16.2)	965 (16.5)
Creatinine, median (IQR), mg/dL (range, 0.4-12. mg/dL)	0.9 (0.8-1.1)	0.9 (0.8-1.1)
eGFR, median (IQR), mL/min/1.73 m^2^ (range, 4-142 mL/min/1.73 m^2^)	86 (71-100)	84 (70-99)
eGFR threshold, mL/min/1.73 m^2^, No. (%)		
≥90	2222 (43.3)	2453 (41.1)
60-89	2293 (44.7)	2767 (46.3)
Stage G3 (30-59)	579 (11.3)	717 (12.0)
G3a (45-59)	455 (8.9)	581 (9.7)
G3b (30-44)	124 (2.4)	136 (2.3)
Stage G4 (15-29)	29 (0.6)	31 (0.5)
Stage G5 (<15)	5 (0.1)	5 (<0.1)

Abbreviations: BP, blood pressure; eGFR, estimated glomerular filtration rate; G, eGFR stage; HbA_1c_, hemoglobin A_1c_.

SI conversion factors: To convert HbA_1c_ to proportion of total hemoglobin, multiply by 0.01; and creatinine to micromoles per liter, multiply by 88.4.

^a^ Missing values: HbA_1c_ (n = 127), systolic BP (n = 781), diastolic BP (n = 776).

^b^ Unique patients are first encounters.

^c^ Race and ethnicity were self-reported; 1 category was suppressed due to low cell count (<5).

**Table 2.** Associations Between Patient Characteristics and Chronic Kidney Disease

Characteristic	Unadjusted		Adjusted^a^	
	RR (95% CI)	*P* value	RR (95% CI)	*P* value
**eGFR <60 mL/min/1.73 m^2^**				
Age^b^				
Q2 vs Q1	4.71 (2.88-7.69)	<.001^c^	3.99 (2.40-6.64)	<.001^c^
Q3 vs Q1	11.33 (7.11-18.06)	<.001^c^	9.32 (5.76-15.06)	<.001^c^
Q4 vs Q1	21.95 (13.91-34.64)	<.001^c^	18.62 (11.64-29.78)	<.001^c^
Sex (male vs female)	0.71 (0.60-0.83)	<.001^c^	0.78 (0.67-0.92)	.002^c^
Race (Black vs race other than Black)	1.63 (1.36-1.95)	<.001^c^	1.73 (1.44-2.08)	<.001^c^
HbA_1c_ ≥6.5%	1.34 (0.73-2.47)	.35	1.18 (1.02-1.38)	.03^c^
Systolic BP ≥130 mm Hg	0.76 (0.51-1.14)	.18	0.95 (0.82-1.09)	.43
**eGFR <45 mL/min/1.73 m^2^**				
Age^b^				
Q2 vs Q1	9.94 (2.33-42.35)	.002^c^	8.07 (1.87-34.76)	.005^c^
Q3 vs Q1	24.68 (6.05-100.69)	<.001^c^	17.62 (4.25-73.00)	<.001^c^
Q4 vs Q1	49.69 (12.37-199.57)	<.001^c^	35.35 (8.66-144.29)	<.001^c^
Sex (male vs female)	0.87 (0.63-1.21)	.42	1.08 (0.77-1.51)	.65
Race (Black vs race other than Black)	1.70 (1.16-2.49)	.006^c^	2.01 (1.31-3.09)	.002^c^
HbA_1c_ ≥6.5%	2.53 (1.82-3.51)	<.001^c^	1.74 (1.25-2.43)	.001^c^
Systolic BP ≥130 mm Hg	1.32 (0.93-1.87)	.12	1.04 (0.75-1.43)	.82
**eGFR <30 mL/min/1.73 m^2^**				
Age^b^				
Q2 vs Q1	3.08 (0.32-29.39)	.33	1.47 (0.31-16.37)	.75
Q3 vs Q1	15.77 (2.08-119.73)	.008^c^	8.66 (1.11-67.44)	.04^c^
Q4 vs Q1	17.53 (2.33-131.91)	.005^c^	11.68 (1.52-89.95)	.02^c^
Sex (male vs female)	1.76 (0.89-3.47)	.10	2.07 (1.04-4.12)	.04^c^
Race (Black vs race other than Black)	2.10 (0.87-5.08)	.10	2.29 (0.89-5.91)	.09
HbA_1c_ ≥6.5%	4.17 (2.17-8.02)	<.001^c^	2.58 (1.30-5.14)	.007^c^
Systolic BP ≥130 mm Hg	2.58 (1.33-5.03)	.005^c^	1.86 (0.94-3.69)	.07

Abbreviations: BP, blood pressure; eGFR, estimated glomerular filtration rate; HbA_1c_, hemoglobin A_1C_; Q, quartile; RR, relative risk.

^a^ Adjusted for age, race and ethnicity, sex, HbA_1c_, and systolic BP categories.

^b^ Age quartiles (Q1, ≤42 years; Q2, 43 to <58 years; Q3, 58 to <68 years; and Q4, ≥68 years).

^c^ Statistically significant.

## Discussion

This is the first report of results from a population-wide screening program using community outreach by MHUs. Findings are similar to national statistics using the CKD-EPI 2021 eGFR creatinine formula showing higher CKD rates among at-risk subgroups—especially older individuals and Black adults.^5^ However, prevalence of CKD stage 3 or higher (based on eGFR alone) in our cohort (11%-13%) was double that of the general US population in the National Health and Nutrition Examination Survey (6.5%) and closer to that in the Veteran’s Affairs cohort (19.6%).^5,6^ This difference is most likely due to our population residing in socially vulnerable communities enriched with CKD risk factors. A limitation was our inability to measure urine albumin to creatinine ratio, which, when added in the future, will result in higher rates of CKD detection. Follow-up eGFR measurements were also not performed to confirm CKD stages, which may result in some misclassification of patients. Altogether, these novel findings suggest significant potential for public health benefits associated with population-wide CKD screening in at-risk communities using MHU platforms.

## Notes

Supplement.
Data Sharing Statement

## References

[1] Kidney Disease: Improving Global Outcomes (KDIGO) CKD Work Group. KDIGO 2024 clinical practice guideline for the evaluation and management of chronic kidney disease. Kidney Int. 2024;105(4S):S117-S314. 38490803 10.1016/j.kint.2023.10.018

[2] Cusick MM, Tisdale RL, Adams AS, . Balancing efficiency and equity in population-wide CKD screening. JAMA Netw Open. 2025;8(4):e254740. doi:10.1001/jamanetworkopen.2025.4740 40227684 PMC11997725

[3] van Mil D, Kieneker LM, Heerspink HJL, Gansevoort RT. Screening for chronic kidney disease: change of perspective and novel developments. Curr Opin Nephrol Hypertens. 2024;33(6):583-592. doi:10.1097/MNH.0000000000001016 39137037 PMC11426989

[4] Twiner MJ, Akcasu NN, Foster B, . Origins of a novel mobile health unit program to prevent cardiovascular disease in vulnerable communities. J Clin Hypertens (Greenwich). 2024;26(4):448-450. doi:10.1111/jch.14800 38501742 PMC11007797

[5] Lima NA, Cardozo S, Johnson A, . Accuracy of a novel high-throughput “car blood pressure” measurement protocol. Am J Hypertens. 2025;38(8):534-536. doi:10.1093/ajh/hpaf016 39886990 PMC13358868

[6] Duggal V, Thomas IC, Montez-Rath ME, Chertow GM, Kurella Tamura M. National estimates of CKD prevalence and potential impact of estimating glomerular filtration rate without race. J Am Soc Nephrol. 2021;32(6):1454-1463. doi:10.1681/ASN.2020121780 33958490 PMC8259653

