# Supplementary material for: Screening for Chronic Kidney Disease by Mobile Health Unit Outreach
Source: JAMA Netw Open. 2026 Mar 18;9(3):e262312. doi: 10.1001/jamanetworkopen.2026.2312 (PMC13000635; doi:10.1001/jamanetworkopen.2026.2312)
Supplement: Supplement. — Data Sharing Statement [file jamanetwopen-e262312-s001.pdf]

Data Sharing Statement

Brook. Screening for Chronic Kidney Disease by Mobile Health Unit Outreach. *JAMA Netw Open*. Published March 18, 2026. doi:10.1001/jamanetworkopen.2026.2312

Data
Data available: Yes
Data types: Deidentified participant data
How to access data: Data Availability: Data are available upon reasonable request to the corresponding author
When available: With publication

Supporting Documents
Document types: None

Additional Information
Who can access the data: Researchers whose proposed use of the data has been approved
Types of analyses: Specified research purpose.
Mechanisms of data availability: With a signed data use agreement
